# Long-term treatment of a child with congenital adrenal hyperplasia

**DOI:** 10.1186/1687-9856-2013-S1-P121

**Published:** 2013-10-03

**Authors:** Rogatianus Bagus Pratignyo, Aditiawati

**Affiliations:** 1 Department of Pediatrics Moh. Hoesin Hospital / Sriwijaya Medical Faculty Palembang, Indonesia

## Background

Congenital adrenal hyperplasia (CAH) is one of the autosomal recessive disorder caused by an enzyme deficiency or defect in one of five enzymatic steps which are required for the biosynthesis of adrenal steroid. CAH could be a medical and social crisis, and especially in South Sumatera, Indonesia it is also associated with cultural problems. *Natural history* of CAH without treatment can cause death due to loss of salt crisis unless it can be detected and treated early.

## Objectives

To report case of long-term salt wasting type of CAH patient, deformity of external genetalia which is now called as disorder of sex development (DSD) and failure to thrive.

## Results

A 45 day old baby admitted with symptoms of diarrhea and vomiting since birth. Physical examination found external genital deformity and failure to thrive, with hyponatremia and hyperkalemia. This baby was consulted to pediatric endocrine subdivision and then suspected as a salt wasting type of CAH. Previously, patient was hospitalized with diarrhea and vomiting for 5 days before referral to Moh. Hoesin Hospital Palembang. Patients had frequent vomiting since birth and difficulties in gaining weight. The parents considered that this child sex was a male. Based on laboratory finding, result of the hormone testosterone and 17-OH progesterone level, a *salt wasting* type of CAH is established. Internal genitalia ultrasound, genitography, and chromosome analysis assigned that patient’s sex was a female. A 2 years observation in this patient after discharged was focused on problems of drug availability, growth, ambigus genitalia, electrolyte disorders, even puberty and reproduction. Also, other problems in families such as knowledge, attitude and behaviour of all family members of CAH disease, adherence to visit polyclinic for monitoring the risk of morbidity and mortality, financial limitations that may inhibits early growth intervention in infants with CAH who need optimal nutritional support, parenting pattern in accomodating the patient as a female, and also psychosocial impact.

## Conclusion

Post-treatment patient with CAH should involve a multidisciplinary care. Not only medical and genetic factors which contribute to succed of long-term treatment of patients with CAH, but also good environmental and family factors are required to optimize the growth and development of children with CAH.

